# Predicting spinal column profile from surface topography via 3D non-contact surface scanning

**DOI:** 10.1371/journal.pone.0282634

**Published:** 2023-03-23

**Authors:** Lionel Rayward, Mark Pearcy, Maree Izatt, Daniel Green, Robert Labrom, Geoffrey Askin, J. Paige Little

**Affiliations:** 1 Biomechanics and Spine Research Group, School of Mechanical, Medical and Process Engineering, Queensland University of Technology, Brisbane City, Australia; 2 Sealy of Australia, Wacol, Australia; 3 Wesley Hospital, Auchenflower, Australia; 4 Mater Health Services, South Brisbane, Australia; National University of Ireland Galway, Galway, Ireland, IRELAND

## Abstract

**Introduction:**

3D Non-Contact surface scanning (3DSS) is used in both biomechanical and clinical studies to capture accurate 3D images of the human torso, and to better understand the shape and posture of the spine–both healthy and pathological. This study sought to determine the efficacy and accuracy of using 3DSS of the posterior torso, to determine the curvature of the spinal column in the lateral lying position.

**Methods:**

A cohort of 50 healthy adults underwent 3DSS and Magnetic Resonance Imaging (MRI) to correlate the contours of the external spine surface with the internal spinal column. The correlation analysis was composed of two phases: (1) MRI vertebral points vs MRI external spine surface markers; and (2) MRI external spine surface markers vs 3DSS external spine surface markers. The first phase compared the profiles of fiducial markers (vitamin capsules) adhered to the skin surface over the spinous processes against the coordinates of the spinous processes–assessing the linear distance between the profiles, and similarity of curvature, in the sagittal and coronal planes. The second phase compared 3DSS external spine surface markers with the MRI external spine surface markers in both planes, with further qualitative assessment for postural changes.

**Results:**

The distance between the MRI vertebral points and MRI external spine surface markers showed strong statistically significant correlation with BMI in both sagittal and coronal planes. Kolmogorov-Smirnov (KS) tests showed similar no significant difference in curvature, k, in almost all participants on both planes. In the second phase, the coronal 3DSS external spine surface profiles were statistically different to the MRI external spine surface markers in 44% of participants. Qualitative assessment showed postural changes between MRI and 3DSS measurements in these participants.

**Conclusion:**

These study findings demonstrate the utility and accuracy of using anatomical landmarks overlaid on the spinous processes, to identify the position of the spinal bones using 3DSS. Using this method, it will be possible to predict the internal spinal curvature from surface topography, provided that the thickness of the overlaying subcutaneous adipose layer is considered, thus enabling postural analysis of spinal shape and curvature to be carried out in biomechanical and clinical studies without the need for radiographic imaging.

## Introduction

Three dimensional (3D) non-contact structured light surface scanners capture calibrated images of 3D objects, enabling virtual 3D reconstruction to be created with a high level of accuracy. The technology rapidly projects light onto an object while simultaneously recording images and detecting how the light distorts to patch together a 3D object. This versatile technology has seen applications across a multiplicity of disciplines, with examples including digital preservation of heritage listed buildings and sculptures [1], design of custom apparel [2]; analysing the biomechanics of a golf swing [3]; and in custom designing and reverse engineering car parts [4]. In certain healthcare applications, 3D surface scanning (3DSS) provides an appealing alternative to radiographic imaging due to its affordability, portability, ease of operation, and absence of ionising radiation exposure [5]. Examples of this application include evaluation of biomechanical and postural parameters, such as chest wall deformities [6], asymmetric muscular development [7], and measuring trunk posture for orthotic brace design [8]. An emerging application is the measurement of scoliosis severity by capturing surface topography of the torso to produce an external spinal contour [9]. Even so, it remains in question whether external surface topography can be used to derive meaningful clinical parameters describing the spinal column, and prior studies using surface topography to evaluate spinal deformity parameters have found varying results, with some finding surface metrics do not relate to clinical deformity angles [10] and others finding good correlation between the two modalities [11, 12]. While Yıldırım *et al*. [10] found a strong correlation between surface derived spinal contours and Cobb angle for 42 scoliosis patients, Hong *et al*. [11] noted that surface topography is most reliable for mild and non-progressive curves.

3DSS of the human body is similarly relevant for the study of healthy human biomechanics. Wu *et al*. [12] used 3DSS to capture body shape when creating a predictive model to assess sleep comfort, however, only scanned participants while standing and thus estimated lying alignment from computational modelling. Huysmans *et al*. [13] similarly evaluated the alignment of the spine using a point distribution model to study postural change and sleep ergonomics when lying laterally, however they assessed only 12 participants and only in one lying position. While literature in the field of spinal deformity shows many examples of the use of 3DSS to interpret spinal curvature (as per Kandasamy *et al*. [14]), there is less evidence for the validation of 3DSS to ensure the use of topographically selected points on the spine accurately represents the 3D position of the vertebrae and spinal curvature. A necessary baseline for research exploring spine biomechanics and posture for healthy participants, as well as for research exploring spinal pathology (eg. spinal deformity), is to determine the accuracy with which the position of surface markers placed over the spinous processes can be used as a surrogate for the position of the spinal bones (ie. Spinous processes). A preliminary study by Little *et al*. [9] compared magnetic resonance imaging (MRI) derived spinal column curvature, MRI derived surface topography curvature and 3DSS derived surface topography curvature in ten healthy female subjects but only for lateral lying. Comparisons were performed in the sagittal plane, and good agreement was observed across the modalities for seven subjects. While an important step in understanding the feasibility of 3DSS to quantify spinal parameters, the study was limited by a small sample size and absence of coronal plane comparison between the externally visible spinal contour and the internal curvature of a line joining the spinous processes. Patias *et al*. [15] highlighted the importance of spinal imaging in all anatomical planes to produce clinically relevant biometrics. The current study sought to further elucidate the efficacy and accuracy of using 3DSS of the external spinal contours to measure the anatomical curvature of the bony spinal column. The study builds upon the previous work from Little *et al*. [9] with a larger cohort of 50 healthy subjects, to compare the accuracy of contours measured from 3DSS and MRI, when viewed in both the coronal and sagittal planes.

## Materials and methods

Institutional human ethics approval was granted from Chair of the University (QUT) Human Research Ethics Committee, in accordance with the National Statement on Ethical Conduct in Human Research (HREC #1700000335). In keeping with this approved protocol, all volunteers provided signed, informed consent to participate in this study. A cohort of 50 adults underwent 3DSS and MRI, where the 3DSS measured the external spine surface contour and the MRI measured both the external spine surface contour and the internal spine contour. Firstly, the external spine surface contour was demarcated using fiducial markers adhered to the skin over the spinous processes. The fiducial markers were vitamin capsules found to be clearly visible in the MR images [16]. The positions of the fiducial markers and spinous processes were then identified from MRI to calculate a *fiducial marker profile (FMP)* and the *spinal column profile (SCP)*. The relationship between the two profiles was analysed in terms of linear distance and difference in curvature between the two profiles. Secondly, 3DSS captured the position of stickers adhered to the skin over the spinous processes to calculate a *3DSS marker profile (3MP)*. The 3MP and FMP were compared to gauge the accuracy of 3DSS in obtaining positional information describing the internal spinal curvature.

### Subjects

The study cohort included 25 men and 25 women of age (mean ± SD) 23.48 ± 2.79 with no history of spinal pathology (Institutional HREC #1700000335). Each participant had height and weight measured to calculate *body mass index (BMI)*:

$$BMI=\frac{\left[weight\;\left(kg\right)\right]}{{\left[height\;\left(m\right)\right]}^{2}}$$

Participants were classified as *underweight* (BMI<18.5), *healthy weight* (BMI:18.5 to 24.9) and *overweight* (BMI: 25 to 29.9) (The Heart Foundation, Australia). BMI was 22.82 ± 3.22 with 15 overweight, 3 underweight, and 32 healthy weight participants. All participants were provided with sport shorts, and females additionally wore a sports bra providing an unimpeded view of the posterior torso surface, particularly the spinal mid-line.

### Imaging

#### 3DSS

**Fig 1:**
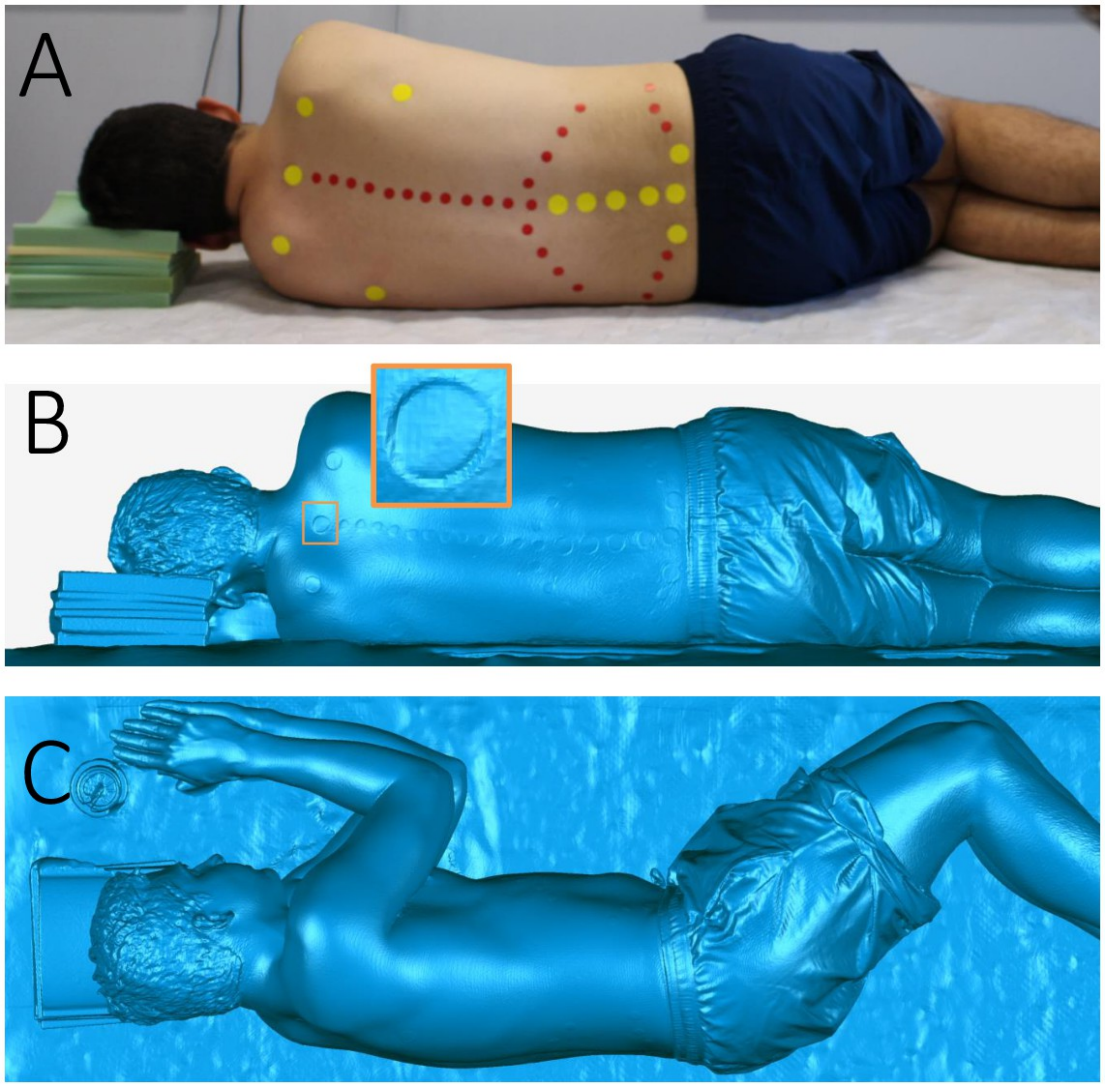
Participant lying laterally during 3DSS. Participant 43 lying laterally on a rigid substrate with a white mat overlaid. The participant’s head is supported with stacked foams and various anatomical landmarks are demarcated with red and yellow stickers. (A) Participants were guided in assuming a posture with the shoulders and hips vertically aligned. (B) The coronal plane of the 3DSS reconstruction is viewed perpendicularly to the substrate surface, with a magnified view of the C7 marker. (C) The sagittal plane is viewed parallel to the substrate surface, oriented such that the line between the origin and L1 marker is horizontal.

3DSS of the participant was performed on a rigid substrate in the lateral position, with surface markers in the form of circular stickers demarcating the spinal column. The circular stickers (diameter: 14 mm) were applied by an experienced physiotherapist (author, MTI) to demarcate the bony spinous processes in the thoracic and lumbar spine with the patient lying prone. The participant was positioned in the left lateral decubitus position, with foam stacks to support the participant’s head so that neck muscle strain was minimised. (Note: This lateral position is not routinely used for spinal imaging, but permitted an unobstructed visibility of these participant’s spine, for comparison of clinical images and topographic images from 3DSS.) The authors instructed the participant in making postural adjustments to ensure the shoulders and hips were aligned vertically, the thighs were at an angle of 60 degrees to the torso, the shanks were at 60 degrees to the thighs, and the upper arms were at 30 degrees to the torso with hands positioned in front of the face (Fig 1). Once positioned, the participants underwent 3DSS using an Artec Eva (Artec3D, Luxembourg City, Luxembourg) non-contact surface scanner to capture the complete torso anatomy. Participant scans took approximately 80–120 seconds, which was a sufficiently short time for the participant to remain stationary for the duration of image capture. The Artec Eva rapidly projected structured light onto the participant, while simultaneously capturing images of the light’s reflection over the participant. The scan, containing a large quantity of textured 3D images, was saved for further processing.

#### MRI

After 3DSS, the participant underwent MRI scanning on a rigid substrate in the lateral position with fiducial markers adhered to the spinal column. The participant had fiducial markers, in the form of vitamin D capsules, adhered to the 3DSS stickers along the spinal column. Vitamin D capsules were used as they displayed excellent contrast, and image clarity was sufficient for capturing the spinous processes. The participant was then relocated to the gantry of a clinical MRI scanner where they were repositioned with the same postural criterion as was used for the 3DSS. A T1-weighted MRI sequence (3-T Philips Acheiva, Echo time 1.725 ms, Repetition time 3.82 ms, In-plane resolution 0.708 x 0.708 mm, Slice spacing 0.75 mm, Scan duration 12 minutes) was used, capturing a field of view from the lower cervical spine to the femoral head with full skin envelope.

### Image analysis

The MR images in DICOM format were analysed to extract the spinous process and fiducial marker positions using commercial image processing software, Amira (AMIRA 6.0.0, Thermo Fischer Scientific, Oregon, USA). Using the in-built ‘segmentation’ tools, single voxels were selected at the contact of the marker with the skin. Next, single voxels were selected at the most posterior point of the T1 to L5 spinous processes. The single voxels were exported as 2D coordinates in the Sagittal plane, and again as 2D coordinates in the coronal plane.

The 3DSS spine surface markers were identified from 3DSS images to extract the 3DSS marker positions.

The textured 3D images produced by the 3DSS were visualised in Artec Studio 12 Professional (Artec Group, Luxembourg City, Luxembourg), automatically registered, fused to create a 3D surface geometry and exported in stereolithographic (STL) format to Geomagic Wrap (Geomagic Wrap, 3D Systems, North Carolina, USA, Vsn 2017).

The coordinate system was then defined so that the sagittal plane was parallel to the substrate surface and the floor, the coronal plane was defined as perpendicular to the coronal plane, while parallel with a line between the L1 marker and mid-point between the posterior iliac spine markers (Fig 1). 3DSS spine surface marker positions were defined by selecting the surface area of the marker using the *lasso selection tool*, and calculating the centroid. The array of surface marker coordinates was exported in a Comma Separated Values (CSV) file using a *python* macro.

### Data analysis

For each participant, three sets of 2D coordinates were obtained for both the sagittal and coronal planes.

This included:

1. Spinous process positions selected from MRI
2. Fiducial spine surface marker positions selected from MRI
3. 3DSS marker positions selected by centroid from 3DSS

The 3DSS marker positions were transformed to the MRI coordinate system. To convert each set of marker positions into statistically comparable profiles, a 7^th^ order polynomial was fit to each set of position coordinates. *RMSE* was calculated for the polynomial fit compared to the original coordinates. The SCP, FMP and 3MP were calculated using the polynomial, calculating the anterior-posterior coordinates (sagittal) and lateral coordinates (coronal) at 20mm intervals along the cephalo-caudal axis. The SCP was then compared to the FMP in terms of linear distance and difference in curvature between the profiles, and the influence of BMI, age and gender was analysed in a correlation analysis. The 3MP and the FMP were quantitatively assessed in a KS test, and qualitatively assessed for differences in posture between the two modalities.

#### Spinous process and fiducial marker comparison

The SCP and FMP were compared in terms of linear distance by calculating RMSE between the two profiles, and is referred to as *root mean squared distance (RMSD)*. The influence of age, BMI and gender on RMSD in the lumbar and thoracic regions of the spine was investigated in a correlation analysis. The influence of soft tissue artefact, including lateral distortion and variation in thickness of the adipose-skin layer, were assessed by investigating the changes in linear distance between the two profiles. A Kolmogorov–Smirnov (KS) test was utilised to compare the curvature of both profiles along the cephalo-caudal length of the spine. The distance was calculated at each point between the two profiles to assess distortion of the adipose tissue lateral to the spine from the coronal plane, and the influence of the adipose thickness in the sagittal plane. RMSD was calculated to quantify the distance between the two profiles. Correlations between RMSD and BMI, age and gender were evaluated using a *Pearson’s Correlation Matrix* in Jamovi (V2.2.2, The jamovi project, Sydney), calculating the Pearson’s coefficient (r) and statistical significance (p) for each set of variables. The null hypothesis, that no monotonic relationship exists between the two variables, was tested by a significance level of 0.05. To test if RMSD’s differed regionally, further correlations were explored by comparing RMSD’s derived from the thoracic region and lumbar region.

The difference in curvature of the SCP and FMP was then evaluated using a two sample KS test. Curvature, k, was calculated at each 20 mm interval along the cephalo-caudal axis of the spine, quantifying the deviation from a straight line between neighbouring points. This was applied using the Matlab function ‘LineCurvature2D.m’ (v1.3.0.0, © 2011, Dirk-Jan Kroon, University of Twente) which uses the equation for curvature of plane parametric curves:

k=∥y′x−yx′∥x′2+y′232

Where x represents the cephalo-caudal coordinates, and y represents the lateral coordinates in the coronal plane, and antero-posterior coordinates in the sagittal plane. Significance tests of the two profiles on each plane were calculated in IBM SPSS Statistics (Version 23). The null hypothesis, that both samples are from populations with the same distribution, was tested by a significance level of 0.05.

Inter- and intra-observer reliability was calculated for the fiducial marker and spinous process positions. Intra-observer reliability was calculated from spinous process and fiducial marker selections from MRI of participant 43 (male, overweight) taken on two occasions, two months apart. Interobserver error was calculated using selections of spinous process and fiducial marker selections from MRI of participant 5 (female, underweight), comparing current selection positions with selections from a co-author approximately one year prior [9]. Reliability was evaluated by determining the Intraclass Correlation Coefficients (ICC), comparing the antero-posterior, lateral and cephalo-caudal coordinates of the point selections. The ICC was evaluated in SPSS to determine the level of agreement, where an ICC value greater than 0.81 is ‘almost perfect agreement’ [17].

#### Fiducial marker profile and 3DSS marker profile comparison

The FMP and 3MP were also compared using a two sample KS test, this time comparing the coordinates, rather than curvature profile. Further qualitative analysis was performed by creating a 3D reconstruction of the skin envelope of the subject in Amira, and visually compared against the 3DSS reconstructions for changes in posture.

## Results

RMSE comparing the positional data of the spinous processes, fiducial markers and 3DSS markers to their resulting 7^th^ order polynomial profiles (3MP, FMP and SCP) were relatively low in both planes. In the sagittal plane RMSE’s were very low for all profiles with (mean ± SD) 0.44 ± 0.30 mm for the 3MP, 0.75 ± 0.29mm for the FMP, and 0.85 ± 0.14 mm for the SCP. In the coronal plane, RMSE’s ranged from 0.72 ± 0.25 mm for the 3MP, 0.92 ± 0.37 mm for the FMP, and 0.91 ± 0.27 mm for the SCP indicating that the spinous process profile can be accurately mapped using 7^th^ order polynomials in both planes.

### Fiducial markers vs spinous processes

Comparison of the SCP with the FMP showed that the fiducial markers were at a distance posterior to the spinous processes with a RMSD of 21.85 ± 6.75 mm. In the coronal plane the fiducial markers were laterally displaced from the spinous processes with an RMSD of 8.51 ± 3.24 mm. The fiducial markers were positioned in the left lateral direction of the spinous processes in 98% of subjects, with an average point to point left lateral distance of 5.17 ± 2.83 mm.

**Fig 2:**
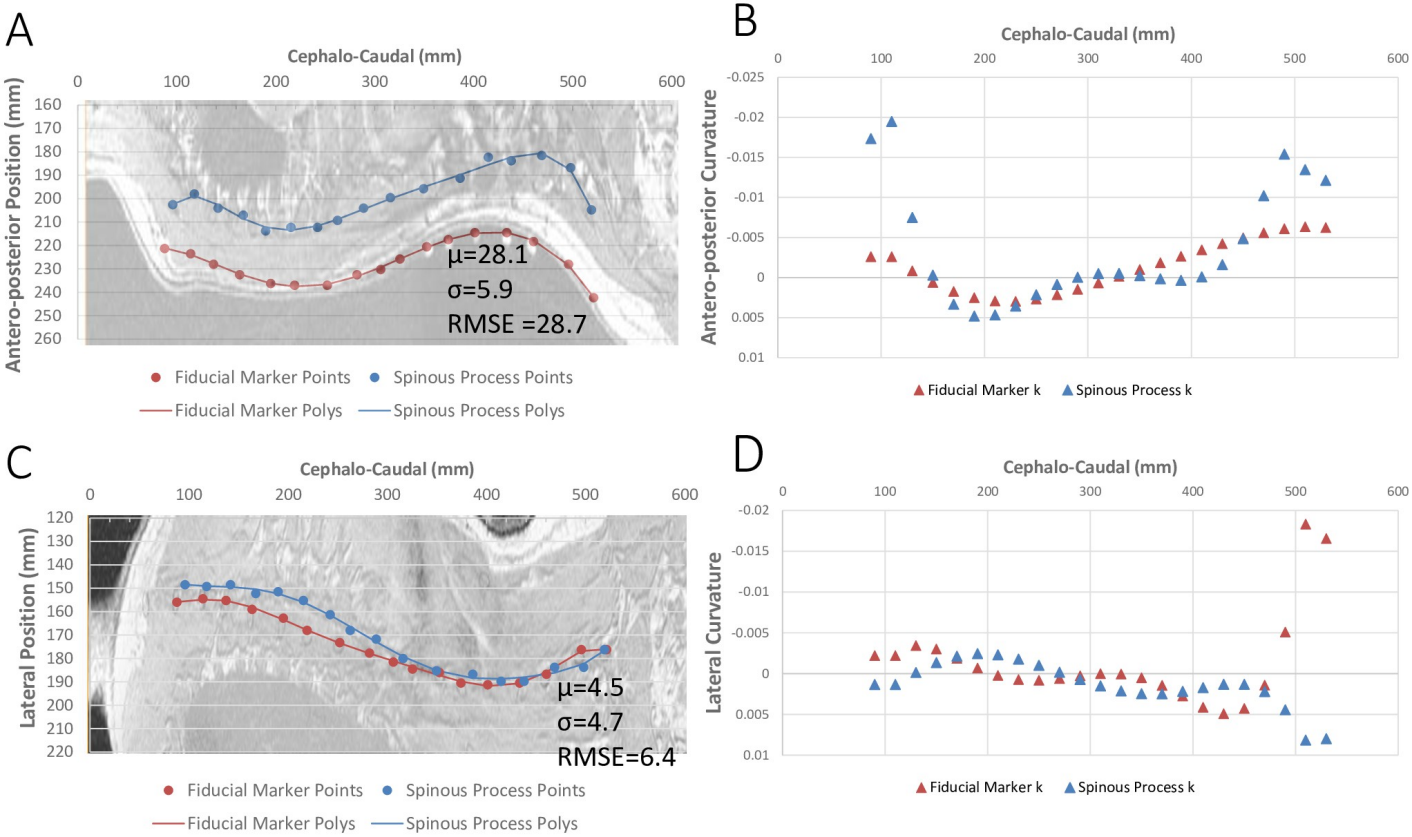
Position and curvature of spinous processes and surface fiducial markers. Position of fiducial markers (Red) and spinous processes (blue) compared in the sagittal (A) and coronal (C) planes with underlaid MR images (rescaled to the plot scale and aspect ratio). (A) The sagittal plane is displayed from the right lateral view, where the cephalo-caudal direction is displayed on the x-axis, and the antero–posterior direction on the y-axis. Participant 17 (shown) is used as an example of low RMSE with intersection in the coronal plane, and consistent distance in the Sagittal plane. (C) The coronal plane is displayed from the posterior view, where the cephalo-caudal direction is displayed on the x-axis, and left lateral direction is displayed on the y-axis. The curvature, k, in the sagittal view (B) and coronal view (D) are shown. μ = mean distance between profiles, mm; σ = standard deviation for the mean distance, mm; RMSE = root mean squared error, mm.

Qualitative assessment of the two profiles showed the antero-posterior distance between the profiles remained consistent in most cases, as the fiducial markers are always posterior to the spinous processes. The direction of lateral distance between profiles was not consistent, with the profiles intersecting for 15 participants in the coronal plane (Fig 2).

**Fig 3:**
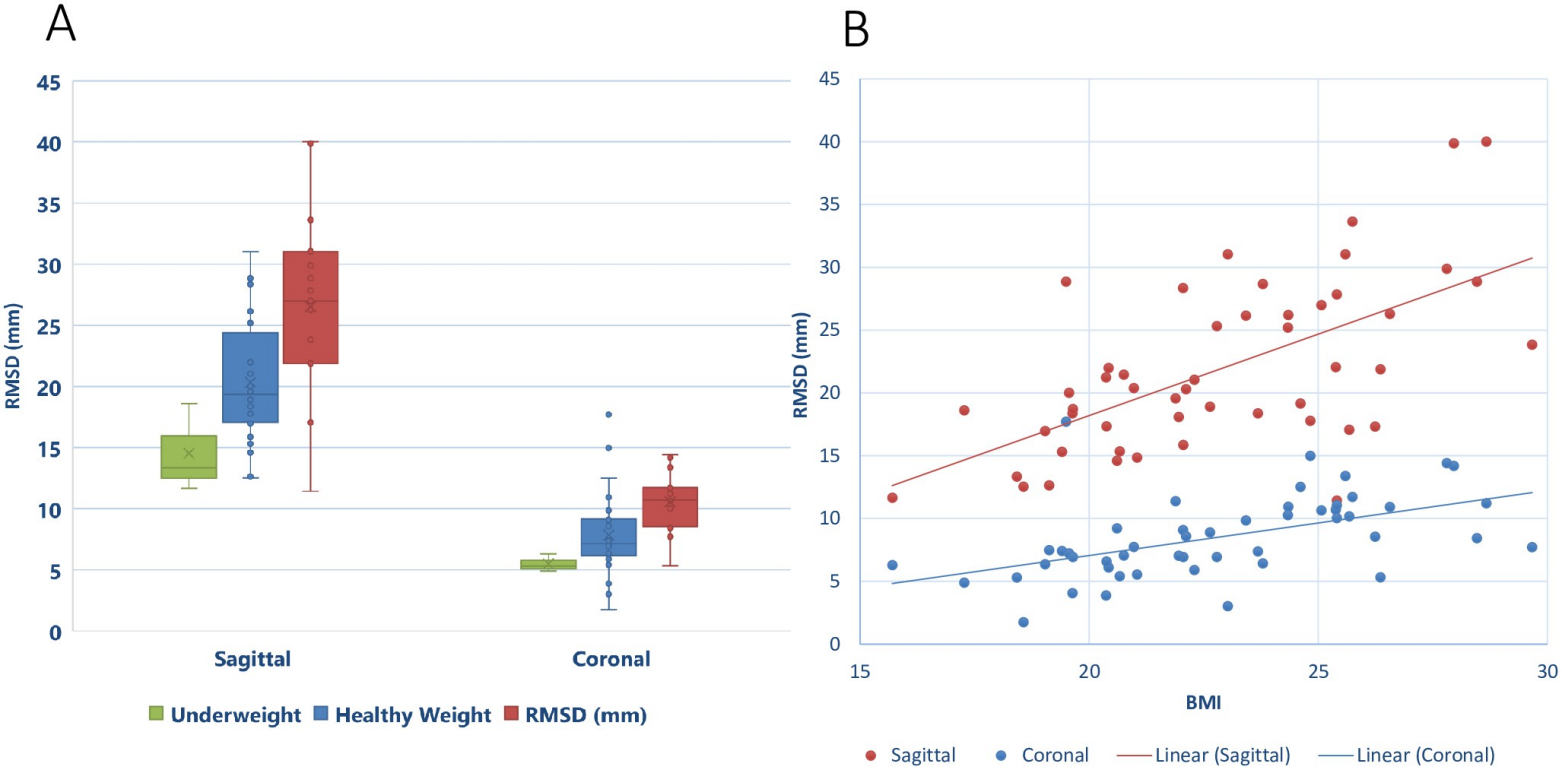
Comparison of the influence of BMI on the fiducial marker—Spinous process RMSD between the coronal and sagittal planes. The y–axis shows the RMSD between the spinous process profile and surface fiducial marker profile within each plane of view. (A) shows a box-plot with underweight (n = 3), healthy weight (n = 32) and overweight (n = 15) BMI categories for each plane. (B) shows a scatter-plot with the BMI of each participant in the x-axis, where the overall RMSD for the sagittal (red) and coronal (blue) is plotted and a linear trendline is fitted. Both the sagittal and coronal views show an increase in RMSD with BMI (B), in the sagittal view it appears BMI also increases the standard deviation (A).

**Fig 4:**
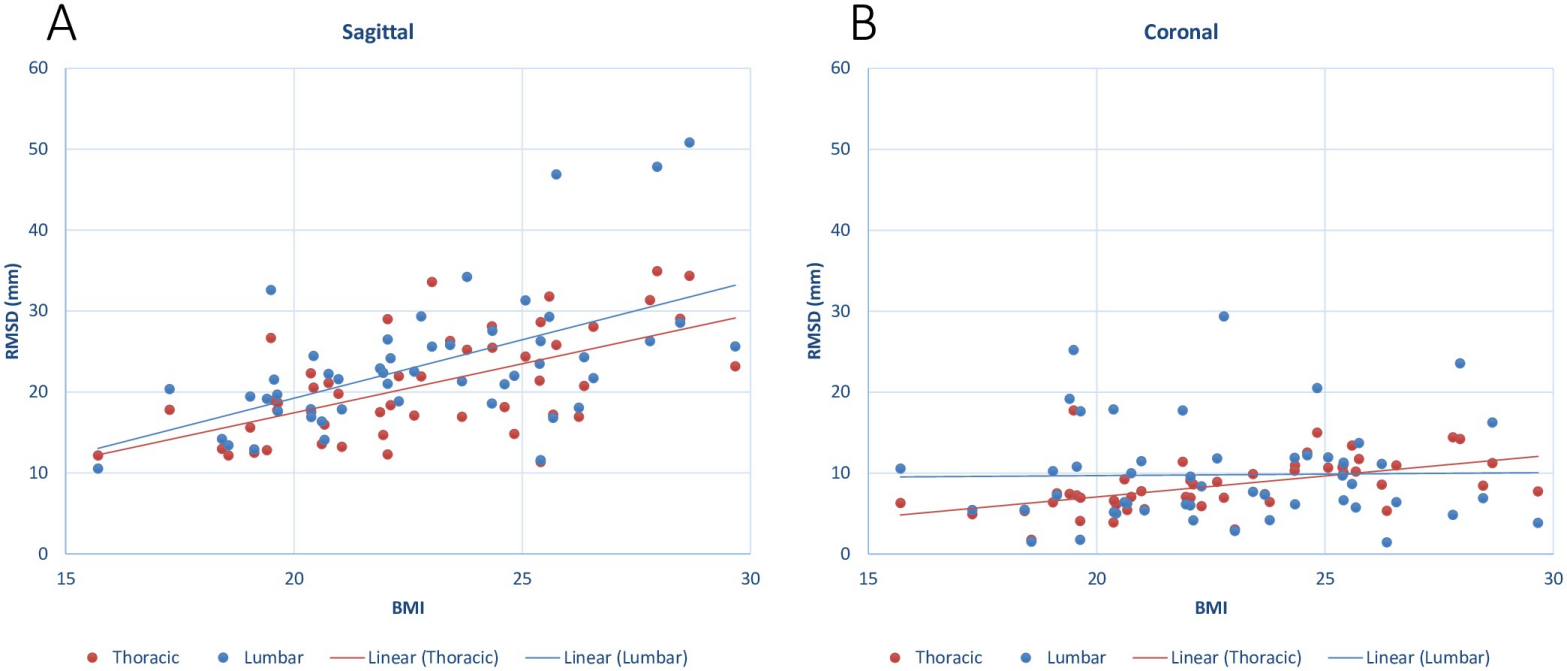
Comparison of the influence of BMI on the fiducial marker—Spinous process RMSD between the thoracic and lumbar regions. The y-axis shows the RMSD between the SCP and the FMP within the specified spinal region of each participant. The x-axis shows the BMI of the participant. The thoracic (red) and lumbar (blue) region RMSE’s are fitted with a linear trendline in both the coronal and sagittal views. In the sagittal view (A), RMSD tends to increase with BMI in both the thoracic and lumbar regions. In the coronal view (B), the thoracic RMSD tends to increase with BMI, where as the Lumbar RMSD maintains a horizontal trend.

Increased BMI resulted in greater linear distances between the two profiles, with an increase in lateral RMSD from underweight to overweight of 76.4% in the coronal plane, and 82.8% in the sagittal plane (Fig 3). Furthermore, strong positive correlations were found between BMI and overall RMSD in both the coronal (Pearson’s r = 0.514, p < 0.001) and sagittal planes (Pearson’s r = 0.597, p < 0.001). Within each region of the spine, lumbar sagittal RMSD (Pearson’s r = 0.558, p < 0.001), thoracic sagittal RMSD (Pearson’s r = 0.597, p < 0.001) and thoracic coronal RMSD (Pearson’s r = 0.533, p < 0.001) all demonstrated strong positive correlation with BMI (Fig 4).

**Fig 5:**
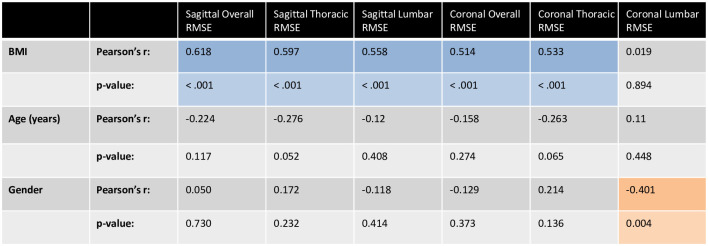
Correlations between BMI, Age, Gender and the FMP to SCP RMSD in the sagittal and coronal planes, in the overall spine (T1 to L5), lumbar spine (L1 to L5) and thoracic spine (T1 to T12). Pearson’s r and p-value calculated in Jamovi are given for each set of correlations. A Pearson’s r value of ±0.5 to ±1 indicates a strong correlation (blue), ±0.3 to ±0.49 (orange) indicates a moderate correlation and ±0.29 to 0 indicates low correlation (grey). The p-value indicates the statistical significance of the correlations, where p < 0.05 indicates the correlation is statistically significant. Gender was analysed such that Female = 1 and Male = 2.

While the lumbar coronal RMSD had no correlation with BMI, it did show moderate correlation with gender (Pearson’s r = -0.401, ρ = 0.004) indicating that in the lumbar region lateral distance between the profiles tended to be higher in female subjects (Fig 5).

**Fig 6:**
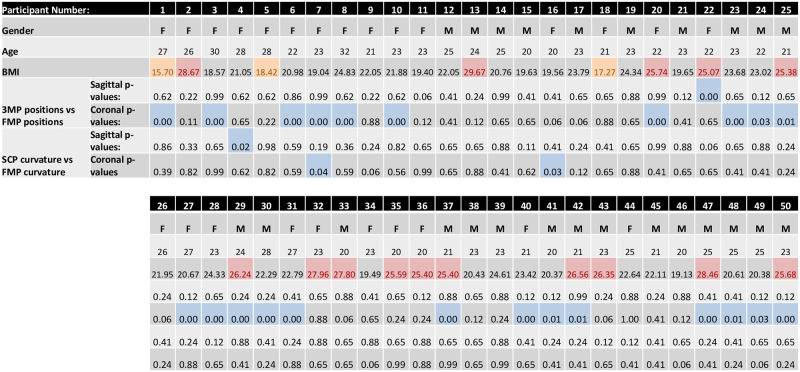
Participant demographics and KS test p-values of 3MP vs FMP and SCP vs FMP. P-values greater than 0.05 indicate no significant difference between the two profiles. Values less than 0.05 (highlighted in blue) indicate significant difference between the two compared profiles. Gender is listed such that F = Female, and M = Male. Age is given as a whole integer at the time of scanning. BMI is classified as underweight (orange), healthy weight, and overweight (red).

**Fig 7:**
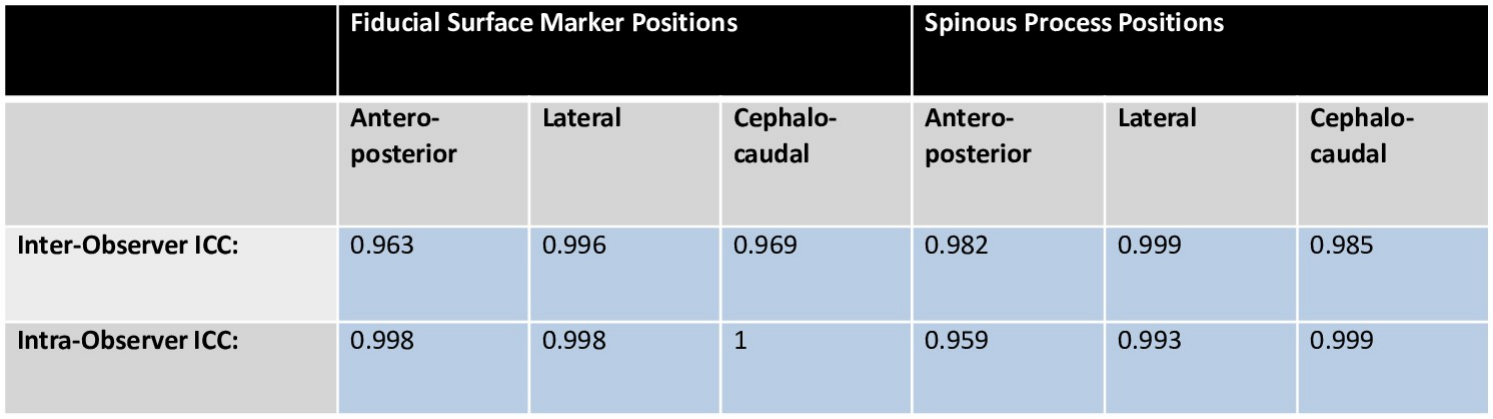
Intra and inter-observer reliability for fiducial surface marker and spinous process position selections. Position data was described by antero-posterior, lateral and cephalo-caudal coordinates, which were each assessed for reliability by calculating ICC. ICC greater than 0.81 describes almost perfect agreement (blue).

KS tests indicated that the curvature distributions of the two profiles in the coronal plane were not significantly different for 96% of participants (KS test p > 0.05) (Fig 6). Similarly, in the sagittal plane the two profiles were not significantly different for 98% of participants (KS test p > 0.05) (Fig 6). In all axes, inter- and intra- observer ICC showed ‘almost perfect’ agreement (ICC > 0.81) (Fig 7).

### Fiducial markers vs 3DSS

In the sagittal plane, FMP and 3MP were not significantly different (KS test p > 0.05) for 98% of participants (Fig 6). In the coronal plane, 54% had no significant difference (KS test p > 0.05) (Fig 6). But when subdividing the population by BMI, in the coronal plane, 67% of the underweight participants, 53% of the healthy weight participants, and 20% of the overweight participants showed no significant difference between the two profiles (KS test p > 0.05) (Fig 6).

**Fig 8:**
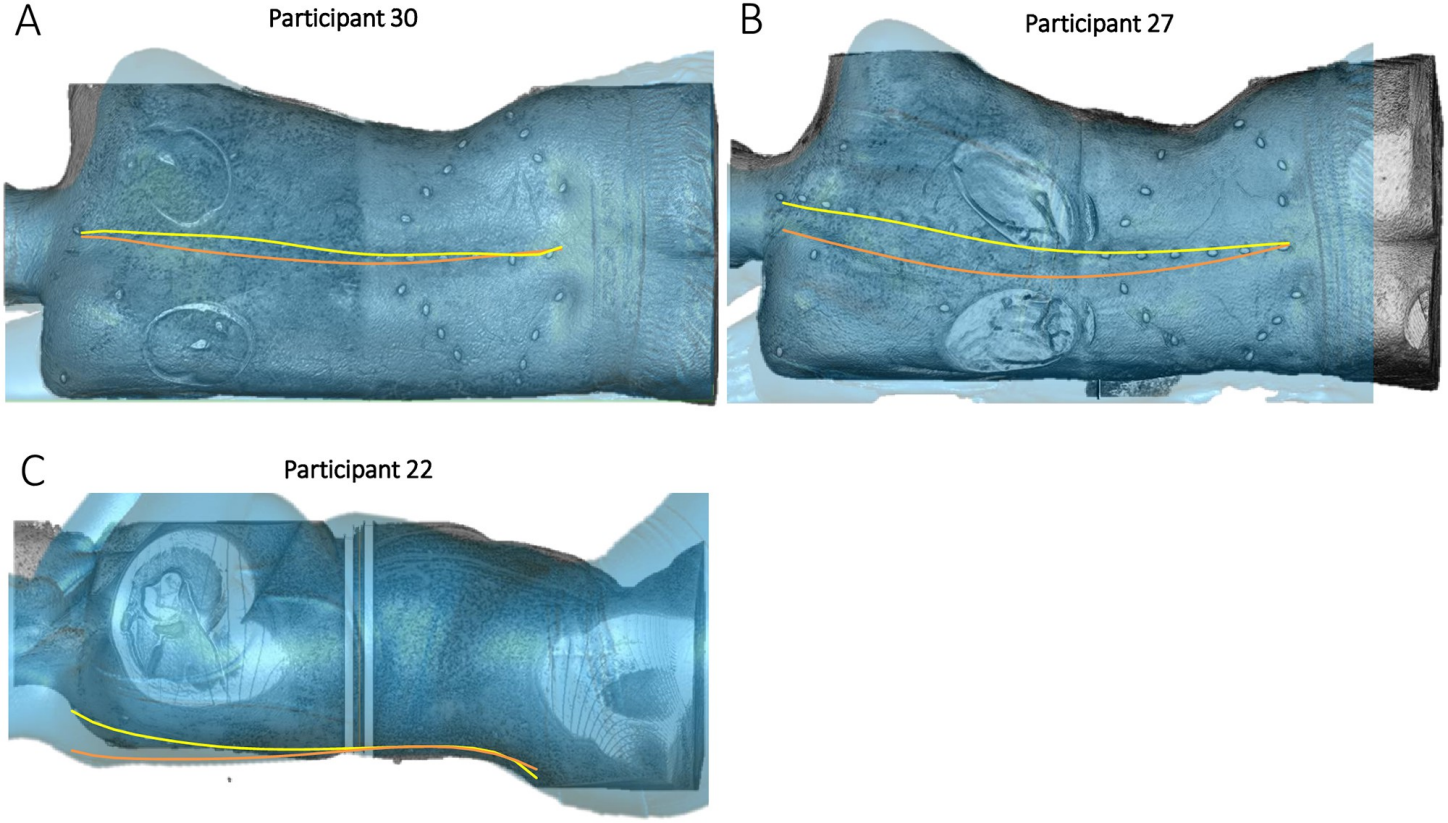
Comparison of the MRI 3D volume render and 3DSS. Comparison of FMP (yellow) and 3MP contours (orange) overlayed on a 3D volume rendering of the MR images (in Amira) shown in grayscale, with the 3DSS shown in transparent Blue (from Geomagic). In comparison to the 3DSS, participant 30 (A), shown in the coronal view, shows elevation of the right shoulder. Participant 27 (B), shown in the coronal view, shows more lumbar lateral flexion in the MRI. Participant 22 (C) shown in the sagittal view, demonstrates more lumbar lordosis and thoracic kyphosis.

Qualitative assessment of the 3D surface reconstructions from the MRI and 3DSS of the participants highlighted postural differences resulting in misalignment of the spinal profiles across the two modalities when comparing the coronal plane. Participant 22, the only participant to have significant differences between the profiles in the sagittal plane (Fig 6), is shown to have different shoulder and hip positions resulting in misalignment of the spinal profile (Fig 8C). Participant 30 and 27 are shown as examples of dissimilar profiles in the sagittal plane, where again there is either a misalignment of the hips or shoulders (Fig 8A and 8B).

## Discussion

The study sought to determine whether fiducial markers on the skin surface, demarcating the spinous processes, could accurately map the internal spine contour of the vertebral bones (spinous processes) in both the sagittal and coronal plane. Additionally, the study sought to demonstrate the feasibility of using 3DSS to map the external spine contour, therefore providing a low-cost radiation free alternative for radiographic imaging. The current study extended on the work of Little *et al*. [9], presenting a study with the addition of analyses in the coronal plane, and a larger cohort allowing for further statistical analysis.

The relationship between the externally visible spinal contour and internal spinal column is of great interest; both in the study of healthy spinal posture, and biomechanics, and in measurement of spinal deformity using externally visible spinal anatomy (eg. Cobb angle). Albeit for the latter of these, 3DSS is most reliable for less severe and/or non-progressive scoliosis deformity [11], since interpreting the internal orientation of scoliotic vertebra is difficult when based on only topographically visible spinal anatomy (ie. spinous processes). To further understand the relationship between external and internal spinal alignment, the current study compared differences in curvature (k) and RMSD between FMC and SCP, using surface markers positioned on the skin overlaying the spinous processes. In keeping with the findings of Goldberg *et al*. [18], this study found no significant difference between surface marker and spinous process curvature in the majority of participants, indicating that it may be possible to predict spinal curvature from surface topography. A comparison of both curvature and position of surface based fiducial markers and MRI-based spinous processes showed that surface markers provide a good representation of both the position of the spinous processes and the curvature of the spine as defined by the processes. This is a critical first step in using surface-based skin markers to derive the posture and alignment of the spine using topographic scanning (ie. 3DSS). Curvature describes the relative changes in spinal posture along the axial length of the spine, and while resolution of surface markers due to artefact introduced from skin and adipose tissue deformation must be considered, the results presented in the current study take this artefact into account in presenting the similarity in *k* and consistency in RMSD between bone and externally located skin markers.

The correlation between BMI and overall sagittal and coronal RMSD confirmed that greater BMI resulted in an increased distance between FMP and SCP due to increased thickness of the subcutaneous adipose layer, and increased magnitude of lateral distortion of the adipose layer from the spinous processes in lateral lying. This is in keeping with Ghaneei *et al*. [19], who found surface topography predictions of spinal deformity severity were significantly improved by eliminating participants with a BMI over 25 from the dataset. The method they proposed depended largely on asymmetry of the posterior rib cage, rather than using the surface topography overlying the spinal column as proposed in this study. Therefore, given no significant difference in curvature between the profiles in the current study, the spine surface markers palpated by a physician may present a way to predict the contour of the posterior spinal column in patients with BMI over 25, provided the increase in linear distance between the profiles is considered. However, no correlation between BMI and lumbar coronal RMSD was evident despite on average greater adipose thickness over this region, suggesting that the effects of lateral adipose distortion may have been obfuscated by postural differences. Axial rotation of the hips, and lateral tension of the skin resulting from friction with the substrate, may have a substantial influence on the lateral distance between the internal and external spine contour.

We note that comparison of 3DSS marker contours to MRI derived spine surface contours was hampered by difficulties in reproducing the same postural position during 3DSS and MRI scanning, and by the fact that 3DSS/MRI cannot capture spinal shape in a single time step, meaning breathing artefact and inability to maintain a single postural position for the duration of the MRI or 3DSS may cause minor variations between imaging measurements. Repositioning was necessary due to the metal componentry of the Artec Eva, which could not be used in the MRI room. Therefore, this study indicates the difficulties in reproducing similar lying posture, even with instruction and oversight. In the sagittal plane, the participants could reproduce the same spinal posture reliably, with only one participant showing significant differences between the spinal contours in this plane. However, in the coronal plane several participants had significant differences in spinal alignment between 3DSS and MRI, indicating a particular sensitivity within this plane to postural changes. Small postural changes tended to shift the position of the L1 vertebra, which was the origin on which all other coordinates were based. Studies attempting to reproduce the spinal curvature inside and outside of an MRI scanner should have very robust methods for maintaining posture.

## Conclusion

This paper sought to explore the feasibility of using *3D non-contact surface scanning (3DSS)* to predict internal spine contour by comparing *magnetic resonance imaging (MRI)* derived *spinal column profile (SCP) with fiducial marker profile (FMP)*, and FMP with 3DSS derived *3DSS marker profile (3MP)*. The curvature, k, of the SCP and FMP reliably showed no significant difference, indicating that internal spine curvature may be predicted from surface topography. However, predictions would need to consider thickness of the subcutaneous adipose overlaying the spinous processes, and lateral distortion of the adipose layer. The 3DSS technique employed in this study has the capacity to capture precise surface topography data, as suggested by reliability of sagittal plane comparisons with MRI data. Significant differences between 3MP and FMP in the coronal plane highlighted the difficulties in reproducing posture in the lateral lying position.
